# Efficacy and Safety of Nivolumab and Ipilimumab for Advanced or Metastatic Renal Cell Carcinoma: A Multicenter Retrospective Cohort Study

**DOI:** 10.3390/curroncol28020133

**Published:** 2021-04-03

**Authors:** Koji Iinuma, Koji Kameyama, Kei Kawada, Shota Fujimoto, Kimiaki Takagi, Shingo Nagai, Hiroki Ito, Takashi Ishida, Makoto Kawase, Kota Kawase, Chie Nakai, Daiki Kato, Manabu Takai, Keita Nakane, Takuya Koie

**Affiliations:** 1 Department of Urology, Graduate School of Medicine, Gifu University, Gifu 5011194, Japan; kiinuma@gifu-u.ac.jp (K.I.); buki21211128@gmail.com (M.K.); stnf55@gmail.com (K.K.); chie.johha@gmail.com (C.N.); andreas7@gifu-u.ac.jp (D.K.); takai_mb@gifu-u.ac.jp (M.T.); keitaco@gifu-u.ac.jp (K.N.); 2 Department of Urology, Kizawa Memorial Hospital, Minokamo 5058503, Japan; i2001029@yahoo.co.jp; 3 Department of Urology, Gifu Prefectural General Medical Center, Gifu 5008717, Japan; keinedvedon@yahoo.co.jp; 4 Department of Urology, Ogaki Municipal Hospital, Ogaki 5038502, Japan; f19533612@gmail.com; 5 Department of Urology, Daiyukai Daiichi Hospital, Ichinomiya 4918551, Japan; kimiaki_takagi5619@yahoo.co.jp; 6 Department of Urology, Toyota Memorial Hospital, Toyota 4718513, Japan; shingo-nagai@nifty.com; 7 Department of Urology, Matsunami General Hospital, Hashima-gun 5016062, Japan; seanoel2@gmail.com; 8 Department of Urology, Gifu Municipal Hospital, Gifu 5008513, Japan; justaskaxis@gmail.com

**Keywords:** metastatic renal cell carcinoma, nivolumab, ipilimumab, immune-oncology treatments, platelet-to-lymphocyte ratio

## Abstract

We conducted a multicenter, retrospective study to evaluate the efficacy and safety of combination nivolumab plus ipilimumab (NIVO+IPI) in 35 patients with advanced or metastatic renal cell carcinoma (mRCC). In this study, we focused on patients who received NIVO+IPI and were stratified into intermediate- or poor-risk disease according to the International Metastatic Renal Cell Carcinoma Database Consortium model at five institutions in Japan. The primary endpoint was overall survival (OS). Secondary endpoints were disease control rate (DCR), best overall response (BOR), objective response rate (ORR), and progression-free survival (PFS). In addition, we evaluated the role of inflammatory cell ratios, namely neutrophil-to-lymphocyte ratio (NLR) and platelet-to-lymphocyte ratio (PLR), as predictive biomarkers in patients with mRCC. The median follow-up period was 1 year, and the 1-year OS rate was 95.8%. The ORR and DCR were 34.3% and 80.0%, respectively. According to BOR, four patients (11.4%) achieved complete response. According to NLR stratification, the 1-year PFS rates were 82.6% and 23.7% when the NLR was ≤4.6 and >4.6, respectively (*p* = 0.04). Based on PLR stratification, the 1-year PFS rates were 81.7% and 34.3% when the PLR was ≤188.1 and >188.1, respectively (*p* = 0.033). Although 71.4% of the patients experienced treatment-related adverse events (TRAEs) with NIVO+IPI, only four patients discontinued NIVO+IPI due to grade 3/4 TRAEs. Patients treated with NIVO+IPI as a first-line therapy for advanced or mRCC achieved relatively better oncological outcomes. Therefore, NIVO+IPI may have potential advantages and may lead to a treatment effect compared to those receiving targeted therapies. In addition, PLR >188.1 may be a useful predictive marker for mRCC patients who received NIVO+IPI.

## 1. Introduction

Renal cell carcinoma (RCC) is the 6th most frequently diagnosed cancer in men and the 13th most common cancer in women in Japan [1]. Although most RCC cases are globally found as an incidental tumor on imaging, survival is highly dependent on the stage at diagnosis, with a 5-year relative survival of only 12% for stage IV metastatic disease [2]. About one-third of cases are diagnosed as metastatic RCC (mRCC), and 20–50% of patients with RCC who undergo surgery will develop metastatic disease. Initial management for stage IV RCC varies according to prognostic factors [3]. First-line targeted therapies with less toxicity and high survival benefits have now become the mainstay of treatment for mRCC [4]. The currently recommended first-line target therapy options in the National Comprehensive Cancer Network guidelines are single-agent tyrosine kinase inhibitors, vascular endothelial growth factor (VEGF) inhibitors, including pazopanib, sunitinib (SUN), axitinib (AXI), and cabozantinib, or everolimus (EVL) and temsirolimus, as mammalian targets of rapamycin pathway (mTOR) inhibitors [2]. However, only 50% and 20% of the patients with mRCC received second- or third-line treatment after treatment using these drugs, respectively [5].

A commonly used, validated model to assess prognosis was developed by the International Metastatic Renal Cell Carcinoma Database Consortium (IMDC) [6]. The IMDC model classified patients with advanced or mRCC into three groups, namely favorable-, intermediate-, and poor-risk groups, using clinical and laboratory risk factors [6]. Approximately 75% of patients with advanced or mRCC are in the intermediate- or poor-risk group and have worse oncological outcomes than those in the favorable risk group [6].

In the CheckMate 025 trial, NIVO (programmed cell death protein 1: PD-1), which is one of immune checkpoint inhibitors (ICIs), was compared to EVL in patients with clear cell mRCC who previously received anti-VEGF therapy [7]. Patients who received NIVO had more significantly improved overall survival (OS) than those who were administered EVL (hazard ratio [HR], 0.73; *p* = 0.002) [7]. In the CheckMate 214 trial, combination therapy with nivolumab plus ipilimumab (cytotoxic T-lymphocyte-associated protein 4; CTLA-4) (NIVO+IPI) was compared with SUN in first-line clear cell mRCC treatment [8]. In patients with intermediate- or poor-risk disease, according to the IMDC model, the 18-month OS rate was 75% with NIVO+IPI and 60% with SUN, and the median OS was not reached with NIVO+IPI versus 26.0 months with SUN (*p* < 0.001) [2]. Additionally, OS benefits were maintained with NIVO+IPI versus SUN in both intermediate- and poor-risk patients after an extended minimum follow-up of 42 months [9]. Of these, 60 Japanese patients were enrolled in the CheckMate 214 trial (31 and 29 in the NIVO+IPI and sunitinib arms, respectively) [10]. Although OS was not significantly different between the two groups (HR, 0.56; 95% confidence interval, 0.19–1.59; *p* = 0.267) because of the small sample size, Japanese patients treated with NIVO+IPI showed a delayed OS benefit compared with those treated with SUN [10]. In addition, the treatment for metastatic RCC has dramatically changed. In an open-label phase III trial (KEYNOTE-426), advanced RCC patients who received pembrolizumab plus AXI had a significantly longer OS and PFS and higher objective response rate than those who received SUN only [11]. In the phase 3 JAVELIN Renal 101 trial, PFS was significantly longer with avelumab plus AXI than with SUN among patients who received these agents as first-line treatments for advanced RCC [7]. The results of the IMmotion151 trial for untreated metastatic RCC revealed that, in the programmed cell death1-ligand 1 (PD-L1)-positive population, the median PFS was 11.2 months in the atezolizumab plus bevacizumab group versus 7.7 months in the SUN group (*p* = 0.022) [12]. In CheckMate 9ER, nivolumab (NIVO) plus cabozantinib demonstrated superiority over SUN by doubling the PFS time and OS rate and significantly improving OS for advanced RCC [13]. Based on these results, it can be suggested that combination therapy may have several advantages with oncological outcomes in advanced or metastatic RCC compared with NIVO monotherapy.

Therefore, we conducted a multicenter, retrospective cohort study to evaluate the efficacy and safety of combination NIVO+IPI in patients with advanced or mRCC.

## 2. Materials and Methods

### 2.1. Patients

In this retrospective multicenter cohort study, we reviewed the clinical records of patients with advanced or mRCC between August 2018 and March 2020 at 8 institutions in Japan. We focused on the patients who received NIVO+IPI and were stratified into intermediate- or poor-risk groups according to the IMDC risk model [6]. We excluded patients who previously received systemic therapy, including tyrosine kinase inhibitors (TKIs) and VEGF or mTOR inhibitors, and those for whom relevant data was missing in the study. Clinicopathological data included age, gender, height, weight, Eastern Cooperative Oncology Group performance status [14], histology, and data on the level of inflammation, including neutrophil count, lymphocyte count, platelet count, neutrophil-to-lymphocyte ratio (NLR), platelet-to-lymphocyte ratio (PLR), whether the patients underwent surgery, metastatic site, and number of metastases.

### 2.2. Treatment Schedule

Before September 2018, the patients received nivolumab at a dose of 3 mg/kg and ipilimumab at a dose of 1 mg/kg intravenously every 3 weeks. Four courses of NIVO+IPI were undertaken as the induction phase. After the induction phase, the patients were administered nivolumab monotherapy at a dose of 3 mg/kg every 2 weeks as the maintenance phase. After October 2018, the patients received nivolumab at a dose of 240 mg. Treatment continued until disease progression according to radiological evaluation or unacceptable toxicity for treatment-related adverse events (TRAEs).

### 2.3. Patient Evaluation

Baseline evaluations included complete history taking and physical examination; chest, abdominal, and pelvic computed tomography (CT); and magnetic resonance imaging (MRI). Tumor staging was performed according to the staging system defined in the American Joint Committee on Cancer Staging Manual [15].

All patients underwent CT or MRI every 1–3 months until disease progression according to radiological evaluation or treatment discontinuation for TRAEs. Best overall response (BOR) was documented as complete response (CR), partial response (PR), stable disease (SD), or progressive disease (PD) using the Response Evaluation Criteria in Solid Tumors (RECIST) guidelines, version 1.1 [16]. The objective response rate (ORR) was defined as patients with a confirmed best response of CR or PR using RECIST. The disease control rate (DCR) was defined as patients with CR, PR, or SD using RECIST.

NLR and PLR were calculated as the absolute neutrophil count and absolute platelet count divided by the absolute lymphocyte count within the peripheral blood, respectively. The cutoff values for NLR and PLR were defined as the minimal value for (1 − sensitivity)2 + (1 − specificity)2 according to the area under the receiver operating characteristic (ROC) curve [17].

### 2.4. Safety

TRAEs were graded according to the National Cancer Institute Common Terminology Criteria for Adverse Events, version 5.0 [18] and reported between the initiation of NIVO+IPI and at least 100 days after the last administration of ICIs.

### 2.5. Statistical Analysis

The primary endpoint was OS. Secondary endpoints were DCR, BOR, ORR, and PFS. In addition, PFS was evaluated according to NLR and PLR. Data were analyzed using the software JMP 14 (SAS Institute Inc., Cary, NC, USA). The date of the administration with NIVO+IPI was used as the starting point for estimates of OS and PFS. OS was defined as the time from NIVO+IPI commencement to death from any cause. PFS was defined as the time from NIVO+IPI commencement to the disease progression. The Kaplan–Meier method was used to evaluate OS and PFS, and differences were assessed according to clinical variables using log-rank test. Multivariate analysis was performed using a Cox-proportional hazards model. All *p* values were two-sided, and *p* value < 0.05 was considered statistically significant.

## 3. Results

### 3.1. Patients

The demographic data of the enrolled patients are shown in Table 1. A total of 36 patients were treated with NIVO+IPI between August 2018 and March 2020 at eight institutions. In this study, 35 patients were enrolled because 1 patient had missing data. Although 62.9% of the patients were diagnosed clear cell RCC, 12 patients (34.3%) did not undergo renal biopsy. The most metastatic site at diagnosis of mRCC was the lung (19 patients, 54.3%). The median NLR and PLR were 3.5 and 215.6, respectively. Based on ROC analysis, the NLR cutoff was estimated to be 4.6 (sensitivity, 72.7%; specificity, 79.2%) and the PLR cutoff was estimated to be 188.1% (sensitivity, 90.9%; specificity, 54.2%).

### 3.2. Efficacy and Oncological Outcomes

**Figure 1:**
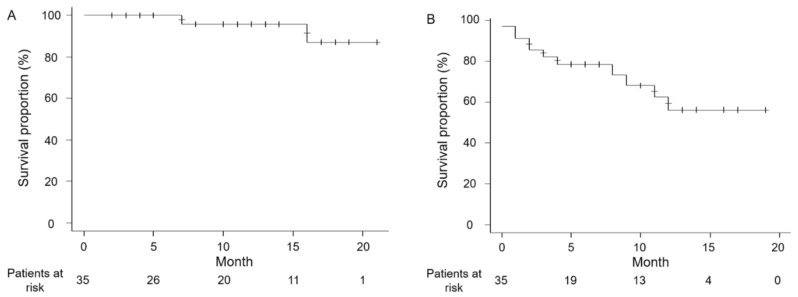
Kaplan–Meier estimates of overall survival (OS) (**A**) and progression-free survival (PFS) (**B**). The OS at 6, 12, and 18 months after nivolumab plus ipilimumab initiation was 100%, 95.8%, and 87.1%, respectively, while the PFS was 78.6%, 56.2%, and 56.2%, respectively.

The OSs at 6, 12, and 18 months were 100%, 95.8%, and 87.1%, respectively (Figure 1A). The PFSs at 6, 12, and 18 months were 78.6%, 56.2%, and 56.2%, respectively (Figure 1B). The median OS and PFS were not reached in this study.

**Figure 2:**
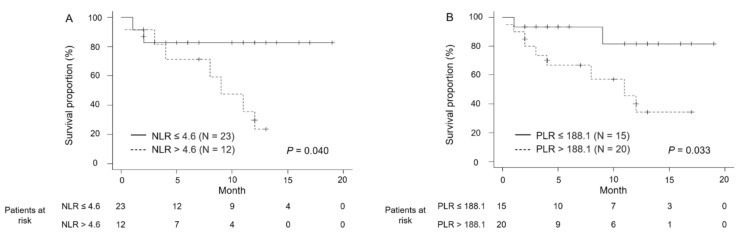
Kaplan–Meier estimates of progression-free survival (PFS) according to neutrophil-to-lymphocyte ratio (NLR) stratified by a cut-off value of 4.6 (**A**) and to platelet-to-lymphocyte ratio (PLR) stratified by a cut-off value of 188.1 (**B**). According to NLR stratification, the 1-year PFS rates were 82.6% when NLR ≤ 4.6 and 23.7% when NLR > 4.6 (*p* = 0.04; Figure 2A). Based on PLR stratification, the 1-year PFS rates were 81.7% when PLR ≤ 188.1 and 34.3% when PLR > 188.1 (*p* = 0.033; Figure 2B).

According to NLR stratification, the 1-year PFS rates were 82.6% and 23.7% when the NLR was ≤4.6 and >4.6, respectively (*p* = 0.04; Figure 2A). Based on PLR stratification, the 1-year PFS rates were 81.7% and 34.3% when the PLR was ≤188.1 and >188.1, respectively (*p* = 0.033; Figure 2B).

The treatment effect in patients who received NIVO+IPI is listed in Table 2. The median follow-up period in this study was 12 (interquartile range (IQR): 4.5–16) months. The median cycles of NINO+IPI was 4 (IQR: 3–4) at the induction phase. The median cycles of nivolumab at maintenance phase was 13 (IQR: 3.5–18). The ORR and DCR were 34.3% and 80.0%, respectively. Three patients (8.6%) discontinued as they developed PD after treatment with ICIs.

In multivariate analysis, IMDC poor risk and PLR >188.1 were associated with poor PFS (Table 3).

### 3.3. Safety

TRAEs are listed in Table 4. A total of 15 patients (42.9%) received steroids (20 mg prednisolone) to manage any grade of TRAEs. TRAEs leading to discontinuation occurred in four (11.4%) patients: Grade 3 hypopituitarism in one patient, grade 4 increased aspartate aminotransferase in one, grade 3 colitis in one, and grade 3 hyperglycemia due to type I diabetes mellitus in one. None of the patients died of TRAEs during the follow-up period.

## 4. Discussion

The clinical characteristics of the patients in this study were generally similar to those of the population of Checkmate 214 [8]. However, the enrolled patients in this study comprised a relatively higher proportion of patients who were classified as IMDC poor risk than those in Checkmate 214 [8]. In this study, ORR and DCR were 34.3% and 80.0%, respectively, for advanced or mRCC patients who received NIVO+IPI. Additionally, 8.6% of the patients who were administered NIVO+IPI achieved CR. Tomita et al. reported that ORR, DCR, and CR rates in a Japanese population are consistent with the results observed in the global population [10]. However, they concluded that further follow-up of the Japanese population may show a late clinical benefit of NIVO+IPI as a first-line treatment for mRCC [10]. Despite the larger population of IMDC poor-risk patients, our results are comparable with these results. Based on the results of Japanese patients in Checkmate 214 and our results, NIVO+IPI as a first-line therapy may have a potential advantage to achieve the treatment effect for advanced or mRCC patients belonging to the intermediate- or poor-risk group, as stratified using the IMDC model.

In the CheckMate 214 trial with a median follow-up period of 25.2 months, NIVO+IPI had significant benefits for OS and PFS [8]. The 12-month and 18-month OS rates were 80% and 75% in patients with NIVO+IPI and 72% and 60% in those with SUN, respectively (*p* < 0.001) [8]. These results demonstrated long-term survival benefits and durable responses with NIVO+IPI after extended follow-up of greater than 42 months [9]. OS and ORR benefits were maintained with NIVO+IPI over SUN in intermediate- and poor-risk patients [9]. Additionally, the PFS had plateaued after 36 months in intermediate- or poor-risk patients who received NIVO+IPI [9]. For these reasons, patients with NIVO+IPI significantly achieved CR more often than those with SUN (*p* < 0.0001) [9]. In addition, almost half the complete responders experienced a treatment-free interval [9]. Therefore, response at 6 months after the initiation of NIVO+IPI may be positively associated with long-term OS for mRCC in the intermediate- or poor-risk groups [9]. However, the Japanese patients treated with NIVO+IPI had a trend toward a later OS benefit than those treated with SUN, even though OS and PFS were similar in the NIVO+IPI and SUN arms [10]. For the demographic and baseline characteristics, Japanese patients who received prior radiotherapy or had high stage disease were relatively lower compared with the global population in the CheckMate 214 trial [10]. These differences may have contributed to the difference of oncological outcomes between the Japanese and global patients [10]. In this study, oncological outcomes, including OS and PFS, were relatively higher than the results of the CheckMate 214 trial. High DCR may be influential in improving oncological outcomes.

Zhang et al. [19] reported that systemic inflammation plays a crucial role in the development and progression of cancer. NLR and PLR can be easily determined from the peripheral blood count. Several studies have evaluated the role of such inflammatory cell ratios as predictive biomarkers in patients with various solid tumors treated with NIVO [19,20,21]. Whereas high baseline NLR and PLR were found to be associated with treatment failure and increased risk of death, low NLR following NIVO treatment was associated with improved oncological outcomes [19,20]. However, the relationship between NLR and PLR and their predictive effects in mRCC patients treated with NIVO+IPI remain unclear. Recently, several meta-analyses have evaluated the utility of PLR as a prognostic factor in cancer patients treated with ICIs [22]. To our knowledge, this is the first study to evaluate the utility of PLR as a prognostic marker in mRCC patients who received ICI therapy, particularly NIVO+IPI. Our study suggests that a high pretreatment PLR may be associated with poor PFS in mRCC patients administered NIVO+IPI. Previous studies have revealed the utility of NLR as an inflammatory biomarker in mRCC patients [23,24,25]. Although NLR was not significantly associated with PFS in the multivariate analysis in our study, mRCC patients with NLR < 4.6 reported a significantly longer PFS than their counterparts. Therefore, NLR may be considered a prognostic marker in mRCC patients treated with NIVO+IPI. Further long-term studies are required to verify the effectiveness of PLR and NLR as prognostic biomarkers in mRCC patients treated with NIVO+IPI.

TRAEs in patients with advanced or mRCC who underwent NIVO+IPI were consistent with that in previous studies for multiple tumor types [26,27], and a relatively lower incidence of grade 3–4 TRAEs was observed than with SUN [8,10,28]. Especially, TRAEs with NIVO+IPI were low grade, and there was a low incidence of grade 3/4 TRAEs compared with targeted therapies, such as SUN, sorafenib, or axitinib [10,28]. Although 15 (42.9%) patients with NIVO+IPI experienced grade 3/4 TRAEs, only 4 patients (11.4%) discontinued NIVO+IPI. However, most Japanese patients may have a manageable safety profile for treatment with NIVO+IPI when combined with a steroid.

There are several limitations to our study. First, this was a retrospective study and was conducted using multicenter data. Therefore, this study had an inherent potential for bias, with diagnostic and therapeutic variations among these institutions. Second, a relatively small number of patients were enrolled in this study, and the follow-up period was relatively short. Third, there was no control group of patients who received TKIs and VEGF or mTOR inhibitors for mRCC. Fourth, we could not evaluate the expression levels of PDL-1 and CTLA-4 because this was a multicenter retrospective study. Indeed, lack of PD-L1 expression correlates with worse outcomes with ICI treatment [29,30]. However, several randomized studies have demonstrated that patients with PD-L1- positive tumors did not show improved OS and ORR [7,8,13]. Finally, we did not collect the duration of response and when the TRAEs occurred during the treatment of NIVO+IPI.

## 5. Conclusions

Although this study was a multicenter retrospective study and a relatively small number of patients was enrolled, patients treated with NIVO+IPI as a first-line therapy for advanced or mRCC may achieve relatively better oncological outcomes. In addition, PLR > 188.1 may be a useful predictive marker for mRCC patients who receive NIVO+IPI. Further studies and long-term evaluations are required to identify the effectiveness of NIVO+IPI, especially in Japanese patients.

## Figures and Tables

**Table 1:** Clinical characteristics of patients.

Number	35
Age (year, median, interquartile range)	69.0 (58.0–76.0)
Gender (number, %)	
Male	26 (74.3)
Female	9 (25.7)
Body mass index (kg/m^2^, median interquartile range)	23.8 (20.7–25.7)
The Eastern Cooperative Oncology Group performance status (number, %)	
0	20 (57.2)
1	7 (20.0)
2	4 (11.4)
3	4 (11.4)
IMDC model (number, %)	
Intermediate-risk	23 (65.7)
Poor-risk	12 (34.3)
Histology	
Clear cell renal cell carcinoma	22 (62.9)
Papillary renal cell carcinoma	1 (2.8)
Unknown	12 (34.3)
Neutrophil counts (×10^9^/L, median, interquartile range)	1.4 (1.1–1.8)
Lymphocyte counts (×10^9^/L, median, interquartile range)	4.5 (3.8–5.6)
Platelet counts (×10^9^/L, median, interquartile range)	265 (213–348)
Neutrophil-to-lymphocyte ratio (median, interquartile range)	3.5 (2.5–4.9)
Platelet-to-lymphocyte ratio (median, interquartile range)	215.6 (138.6–316.3)
The patients who underwent surgery before the administration of NIVO+IPI (number, %)	19 (54.3)
Number of metastatic sites	
0	4 (11.5)
1	11 (31.4)
2	11 (31.4)
≥3	9 (25.7)
Total number of metastatic sites (number, %)	
Lung	19 (54.3)
Lymph node	14 (40.0)
Bone	12 (34.3)
Liver	7 (20.0)
Adrenal gland	5 (14.3)
Pancreas	2 (5.7)
Local recurrence	2 (5.7)
Others	4 (11.4)

IMDC: The International Metastatic Renal Cell Carcinoma Database Consortium; NIVO+IPI: Combination nivolumab plus ipilimumab.

**Table 2:** The treatment effect in patients who received NIVO+IPI and nivolumab.

Number	35
Objective response rate (CR + PR, number, %)	12 (34.3)
Disease control rate (CR + PR + SD, number, %)	28 (80.0)
Best overall response (number, %, 95% CI)	
CR	3 (8.6)
PR	9 (25.7)
SD	16 (45.7)
PD	7 (23.5)

NIVO+IPI: Combination nivolumab plus ipilimumab; CR: Complete response; PR: Partial response; SD: Stable disease; PD: Progression disease.

**Table 3:** Multivariate analysis of clinical parameters for the prediction of progression-free survival.

	*N*	Hazard Ratio	95% Confidence Interval	*p*
Age				
≤69	18	2.38	0.57–9.9	0.233
>69	17	1 (ref.)	-	-
Gender				
Male	26	3.63	0.68–19.3	0.130
Female	9	1 (ref.)	-	-
IMDC risk classification				
Poor	12	6.09	1.08–34.1	0.040
Intermediate	23	1 (ref.)	-	-
Neutrophil-to-lymphocyte ratio				
>4.6	12	2.73	0.58–12.9	0.204
≤4.6	23	1 (ref.)	-	-
Platelet-to-lymphocyte ratio				
>188.1	20	9.46	1.51–58.9	0.016
≤188.1	15	1 (ref.)	-	-

IMDC: International Metastatic Renal Cell Carcinoma Database Consortium.

**Table 4:** Treatment-related adverse events.

Event (Number, %)	Any Grade	Grade 3/4
Treatment-related adverse events	25 (71.4)	15 (42.9)
Hypopituitarism	5 (14.3)	3 (8.6)
Maculopapular rash	5 (14.3)	0
Colitis	4 (11.4)	3 (8.6)
Hypothyroidism	4 (11.4)	2 (5.7)
Pneumonitis	4 (11.4)	0
Arthritis	3 (8.6)	2 (5.7)
Pruritus	3 (8.6)	0
Increased AST	2 (5.7)	2 (5.7)
Increased ALT	2(5.7)	2 (5.7)
Myalgia	2 (5.7)	1 (2.9)
Weight loss	2 (5.7)	0
Hyperglycemia	1 (2.9)	1 (2.9)
Urticaria	1 (2.9)	0
Hyperthyroidism	1 (2.9)	0
Increased creatinine	1 (2.9)	0

ALT: Alanine aminotransferase; AST: Aspartate aminotransferase.

## Data Availability

The data presented in this study are available on request from the corresponding author. The data are not publicly available due to privacy and ethical reasons.
